# Mitochondrial phylogeny within the Yellow Chat (*Epthianura crocea*) does not support subspecific designation of endangered Alligator Rivers population

**DOI:** 10.1002/ece3.9114

**Published:** 2022-07-24

**Authors:** Robin Leppitt, Alea Rose, Wayne A. Houston, Peter M. Kyne, Sam C. Banks, John C. Z. Woinarski, Stephen T. Garnett

**Affiliations:** ^1^ Research Institute for the Environment and Livelihoods Charles Darwin University Casuarina Northern Territory Australia; ^2^ Threatened Species Recovery Hub National Environmental Science Program Canberra Australian Capital Territory Australia; ^3^ Central Queensland University Rockhampton Queensland Australia

**Keywords:** birds, genetic diversity, Meliphagidae, mtDNA, phylogeny, subspecies, taxonomy

## Abstract

The delineation of subspecies is important in the evaluation and protection of biodiversity. Subspecies delineation is hampered by inconsistently applied criteria and a lack of agreement and shifting standards on how a subspecies should be defined. The Australian endemic Yellow Chat (*Epthianura crocea*) is split into three subspecies (*E. c*. *crocea*, *E. c. tunneyi*, and *E. c. macgregori*) based on minor plumage differences and geographical isolation. Both *E. c. tunneyi* (Endangered) and *E. c. macgregori* (Critically Endangered) are recognized under Australian legislation as threatened and are the subject of significant conservation effort. We used mitochondrial DNA to evaluate the phylogeny of the Yellow Chat and determine how much genetic variation is present in each of the three subspecies. We found no significant difference in the cytochrome b sequences (833 base pairs) of *E. c. crocea* and *E. c. tunneyi*, but approximately 0.70% or 5.83 bp difference between *E. c macgregori* and both *E. c. crocea* and *E. c. tunneyi.* This analysis supports the delineation of *E. c. macgregori* as a valid subspecies but does not support separation of *E. c. crocea* from *E. c. tunneyi*. We also found very low levels of genetic variation within the Yellow Chat, suggesting it may be vulnerable to environmental change. Our results cast doubt upon the geographic isolation of *E. c. crocea* from *E. c. tunneyi*, but more advanced genetic sequencing and a robust comparison of plumage are needed to fully resolve taxonomy.

## Graphical abstract

The Yellow Chat *Epthianura crocea* has an unsettled taxonomic history, with particular doubt over the delineation of the threatened *E. c. tunneyi* from *E. c. crocea*. We found no difference in the mitochondrial DNA of these two subspecies, casting doubt on their geographical isolation.

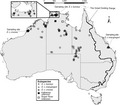

## 1 INTRODUCTION

The taxonomic delineation of subspecies is biologically significant as these represent distinct evolutionary lineages (Lidicker, 1962; Smith & Patton, 1980), and therefore, biodiversity. Despite recent advances in methods to evaluate intraspecific phylogenies, the identification of subspecies often relies on inconsistently applied criteria such as geographic isolation, morphology, life history, behavior, and ecology (Sackett et al., 2014). This is further hampered by the lack of agreement on a universal definition of how taxa are defined (summarized in Garnett & Christidis, 2017). Phylogenetic analysis using DNA has revealed inconsistencies in the boundaries of subspecies that were delineated using less technical means (Zink, 2004). Under Australian legislation, subspecies can be recognized as threatened and the conservation of some threatened subspecies can involve substantial funding and effort (e.g., Department of the Environment and Energy, 2015). Accurate taxonomic delineation of subspecies is therefore important for ensuring that conservation funding is being appropriately allocated to conserve genuine biodiversity.

The taxonomic history of the Yellow Chat *Epthianura crocea* (Aves: Meliphagidae) has been unsettled. Keast (1958) recognized four subspecies (*E. c. crocea*, *E. c. tunneyi*, *E. c. macgregori*, and *E. c. boweri*), based on relatively minor differences in the breeding plumage of males (mainly the brightness of color) and geographic isolation. Subsequently, Ford and Parker (1974) considered it “unwise to treat *crocea* trinomially until more is known of its distribution and movements”. Schodde and Mason (1999) considered that the exact delineation of subspecies was “uncertain” because of limited sampling and labile morphology but accepted three subspecies, incorporating *E. c. boweri* into *E. c. crocea*. Their reasons for sinking *E. c. boweri* are not made completely clear, but they state that *E. c. crocea* is likely a wide ranging and nomadic single population that includes *E. c. boweri*. This delineation was followed by Higgins et al. (2001). However, no subsequent study has examined variation across the species as a whole.

Two of the currently recognized subspecies of Yellow Chat are of conservation concern. The Capricorn or Dawson Yellow Chat *E. c. macgregori* and the Alligator Rivers Yellow Chat *E. c. tunneyi* are listed as threatened under the Australian Government's *Environment Protection and Biodiversity Conservation Act* (EPBC Act) 1999 on the basis of small and declining populations of fewer than 250 individuals (Garnett et al., 2011), although the evidence for these estimates for *E. c. tunneyi* is sparse. Both of these subspecies have been the subject of conservation efforts.

**FIGURE 1:**
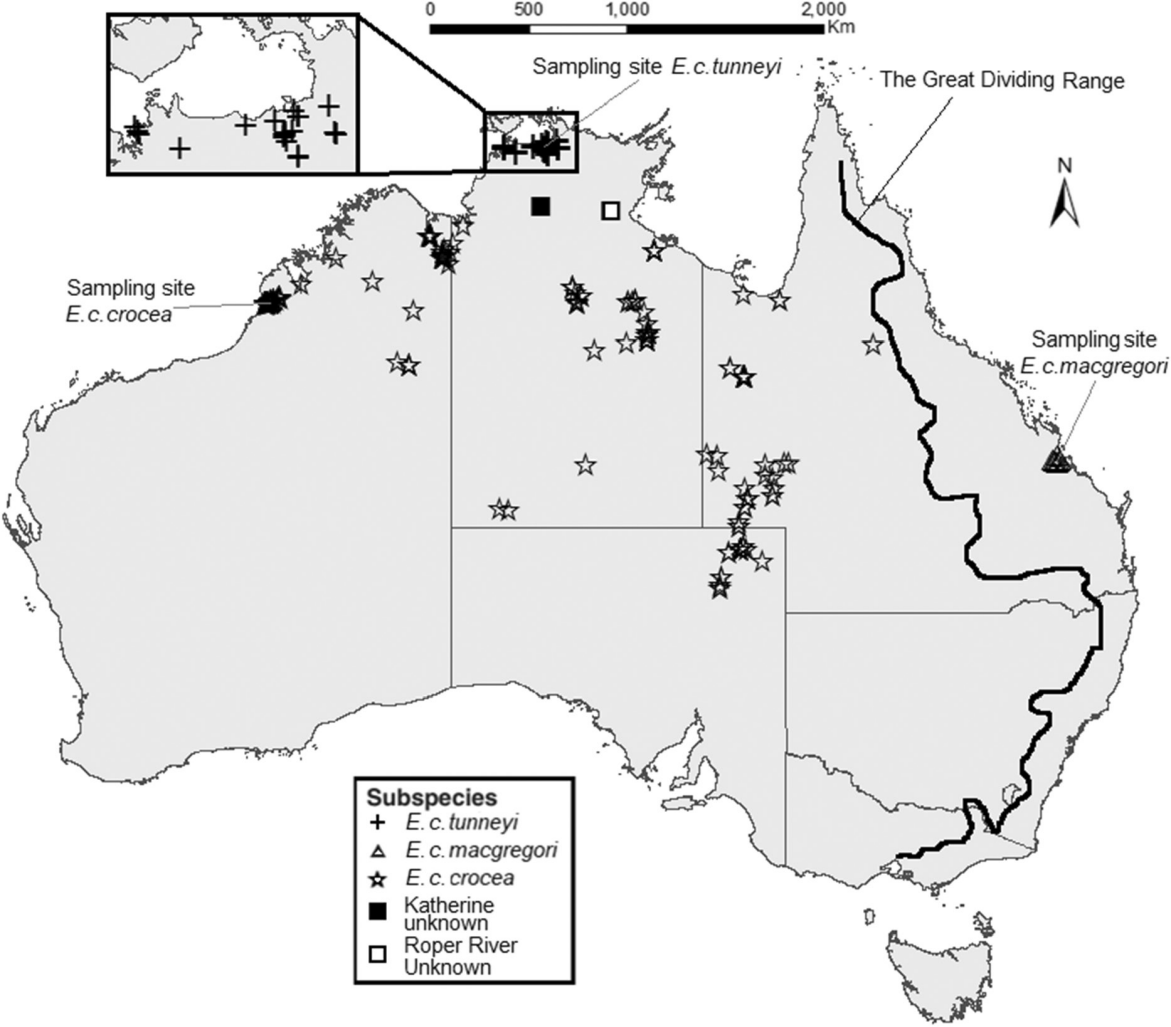
Records of the three subspecies of Yellow Chat (*Epthianura crocea*) sourced from eBird and BirdData records (Birdlife Australia, 2020; eBird, 2021). Broad sampling sites for each subspecies are also shown

The delineation of *E. c. crocea* and *E. c. macgregori* as distinct subspecies is well supported. These two subspecies are geographically separated by ~400 km: *E. c. crocea* inhabits ephemeral wetlands across large areas of central northern Australia, while *E. c. macgregori* only inhabits coastal plains near Rockhampton, Queensland (23.38°S, 150.51°E) (Houston et al., 2013). Between them is the Great Dividing Range, providing an extensive habitat and geographical barrier (Figure 1). Additionally, the two subspecies were found to differ in the mitochondrial cytochrome b gene (Houston et al., 2015). Mitochondrial mutation rates have often been used to separate bird lineages, with populations developing an increasingly different set of haplotypes the longer they are genetically isolated (Weir & Schluter, 2008). Populations that have genuine geographical isolation therefore often differ in their mitochondrial DNA. A study involving the mtDNA of 18 *E. c. macgregori* and 1 *E. c. crocea* found a divergence in the cytochrome b sequence of 4 substitutions or 0.43% between *E. c. macgregori* and *E. c. crocea* (Houston et al., 2015), compared to no variation detected within *E. c. macgregori*, suggesting an extensive period of isolation.

The delineation of *E. c. crocea* and *E. c. tunneyi* as distinct subspecies has not been corroborated by genetic evidence and the geographical distance separating the two subspecies is unclear. The subspecific status of Yellow Chats in Katherine, Northern Territory (14.45°S, 132.27°E; eBird, 2021), reported on July 12–November 11, 2018 and January 4, 2020, ~330 km from the nearest known sightings of *E. c. crocea* on the Victoria River floodplains and ~225 km from the nearest sightings of *E. c. tunneyi* on the South Alligator River floodplains, is unknown. The same is true for an individual seen on the Roper River floodplain (14.67°S, 135.25°E) on November 8, 2018 (eBird, 2021), 190 km from *E. c. crocea* on the McArthur River floodplains and 370 km from the nearest known *E. c. tunneyi* on the East Alligator River Floodplains.

The pattern of Yellow Chat movements is not well characterized. Keast (1958) stated that “taxonomic findings conclusively support distributional evidence that birds breed in the general area of their birth and that there is no interchange of individuals between the four isolated populations” but Higgins et al. (2001) described it as nomadic and dispersive, with the ability to fly long distances to colonize newly suitable habitat. A study of *E. c. macgregori* dispersal found a pattern of small‐scale (<10 km) seasonal movement between breeding habitat and interconnected dry season habitat (Houston, Aspden, et al., 2018). It is therefore possible that *E. c. crocea* and *E. c. tunneyi* are encountering one another, and possibly inter‐breeding.

This study sought to improve our understanding of the genetics, and hence taxonomic resolution, of the Yellow Chat subspecies complex. We used mitochondrial DNA from the cytochrome b loci of all three currently recognized subspecies to: (1) conduct a preliminary evaluation of the phylogeny of the Yellow Chat, in particular the extent of differentiation between *E. c. tunneyi* and *E. c. crocea*, (2) investigate the genetic variation in each of the subspecies, and (3) evaluate the phylogeography of the Yellow Chat. Genetic diversity is important for taxa when dealing with environmental change, as a population with a higher variability of alleles will be better able to evolve and have greater resistance to disease and other stresses (Hughes et al., 2008). This is the first study to examine genetic material from *E. c. tunneyi* and involves a larger sample size of *E. c. crocea* than previous studies (Houston et al., 2015). Greater understanding of Yellow Chat phylogeny will help resolve the subspecific status of the threatened *E. c. tunneyi* and *E. c. macgregori*, while understanding genetic diversity in each of the subspecies can direct future conservation efforts.

## 2 MATERIALS AND METHODS

### 2.1 Sampling

Feathers of *E. c. macgregori*, *E. c. tunneyi*, and *E. c. crocea* were obtained from chats captured using mist nets, with 5–10 chest or belly down feathers from each bird taken for genetic analysis. Feathers have been shown to be a reliable non‐invasive technique for extracting DNA (Taberlet & Bouvet, 1991), and their collection is less invasive than blood extraction.

A total of 21 *E. c. tunneyi* were captured and sampled on the floodplain of the South Alligator River in Kakadu National Park, Northern Territory in November 2017 and 2018. Sampling was undertaken at one location on the western floodplain (12.42°S, 132.37°E) and one location on the eastern floodplain (12.26°S, 132.50°E; Figure 1). For *E. c. crocea*, 11 birds were sampled in November 2019 on Roebuck Plains Station, near Broome, Western Australia (17.97°S, 122.43°E; Figure 1). The feathers of *E. c. tunneyi* and *E. c. crocea* were sampled under Charles Darwin University Animal Ethics Committee permit number A16040, Access to Biological Resources in a Commonwealth Area for Non‐Commercial Purposes permit AU‐COM2017‐350, Government of Western Australia Fauna Taking License FO25000172, and Australian Bird and Bat Banding Scheme (ABBBS) authority 3268. The feathers of 18 *E. c. macgregori* were sampled at two locations (22.60°S, 149.96°E and 23.61°S, 150.73°E) near Rockhampton, Queensland in July and November 2012. The *E. c. macgregori* birds were sampled under Central Queensland University ethics permit A12/02–279, Queensland Scientific Purposes Permit SP08039210, and ABBBS authority A706.

### 2.2 DNA extraction, sequencing, and genotyping

DNA was extracted from chat feathers with a DNeasy® Blood and Tissue Kit from Qiagen® following the manufacturer's protocols with the modifications described in Gebhardt et al. (2009). We used the mitochondrial cytochrome b PCR primers (943 base pair [bp]) YC‐CYB (Forward TTACTAGGCATCTGCTTAACAACC; Reverse TTTTGTTCTCTAGCATGCTTGC) (Houston et al., 2015). We amplified 2 μ L of DNA in a 20 μ L PCR reaction. DNA was amplified using a Labnet MultiGene™ (Labnet International Inc.) under the following PCR conditions: 10 min at 95°C, followed by 50 cycles of 15 s at 95°C and 60 s at 64°C. We checked the PCR product size and concentration of 4 μ L of PCR product using electrophoresis on 2% agarose gel in 1XTBE buffer, stained with GelRed Nucleic Acid dye (Biotium; 10,000 X in water). The gel was run for 70 min at 70 V. Sanger sequencing was outsourced to Macrogen using the same primers used for the PCR. Sequences were trimmed and aligned using the software Geneious Prime 2020 2.2 (https://www.geneious.com).

### 2.3 Analyses

The genetic diversity within each subspecies and the species as a whole was assessed by calculating the nucleotide and haplotype diversities according to Nei (1987) using DnaSP (Rozas et al., 2017). We used the software MEGA 10.1.8 (Kumar et al., 2018) to assess the percentage of pairwise differences of the cytochrome b sequences both within and between the subspecies using a gamma‐distributed maximum composite likelihood estimation with 1000 bootstrap replications. Analysis of molecular variance (AMOVA; Peakall & Smouse, 2006) was used to quantify genetic variation within and among the recognized subspecies in GenAlEx 6.1 and the significance of differentiation (PhiPT) assessed with 999 permutations.

We used PAUP V 4.0 to evaluate the phylogenetic relationships of the Yellow Chat using the unique haplotypes of each subspecies. The Gibberbird (*Ashbyia lovensis*), the sister species to the *Epthianura* chats (Joseph et al., 2014), was used as an outgroup to root the tree (GenBank accession number AY488337; Driskell & Christidis, 2004). Data were analyzed using the maximum likelihood algorithm and pairwise genetic distances among sequences estimated by the general time‐reversible model, GTR + G, in which all models are nested. One thousand bootstrap replicates were evaluated using a heuristic search in PAUP. This incorporates a bootstrap test of the reliability of each node of the tree; nodes with less than 70% reliability are not considered to indicate a reliable separation of those clusters (Hall, 2013).

## 3 RESULTS

Of the samples obtained, sequences of 17 *E. c. tunneyi* (of 21 collected), 8 *E. c. crocea* (of 11 collected), and all 18 *E. c. macgregori* were suitable for analysis. A full 943 bp cytochrome b sequence was obtained for 33 of the birds, while a partial 833 bp sequence was obtained for an additional 10 birds. We included these 10 birds in the analysis to increase our effective sample size, meaning an 833 bp sequence was analyzed for the 43 birds. From these 43 sequences, there were 11 unique haplotypes: 4 from *E. c. tunneyi*, 4 from *E. c. crocea*, and 3 from *E. c. macgregori* (Table 1).

**TABLE 1:** Genetic diversity statistics for each of three Yellow Chat subspecies and for the species as a whole over 833 bp cytochrome b mtDNA sequence

Subspecies	Sample size (*n*)	Haplotypes detected	Polymorphic sites	Haplotype diversity (h)	Nucleotide diversity (*π*)
*E. c. crocea*	8	4	3	0.25	0.00030
*E. c. tunneyi*	17	4	4	0.51	0.00069
*E. c. macgregori*	18	3	2	0.54	0.00074
Yellow Chat (all subspecies)	43	11	9	0.73	0.00378

The average percentage pairwise differences and the average amount of pairwise substitutions of the cytochrome b sequences were greater between *E. c. macgregori* and *E. c. tunneyi* (5.75 bp or 0.69%), and *E. c. macgregori* and *E. c. crocea* (5.83 bp or 0.70%), than it was for *E. c. tunneyi* and *E. c. crocea* (0.42 bp or 0.05%; Table 2).

**TABLE 2:** The average percentage pairwise difference of cytochrome b sequences (gray cells) and the average amount of pairwise substitutions (white cells) between the three Yellow Chat (*Epthianura crocea*) subspecies and the outgroup Gibberbird (*Ashbyia lovensis*)

	*A. lovensis*	*E. c. tunneyi*	*E. c. crocea*	*E. c. macgregori*
*A. lovensis*		68.97	69.06	69.39
*E. c. tunneyi*	8.28%	0.07%	0.42	5.75
*E. c. crocea*	8.29%	0.05%	0.03%	5.83
*E. c. macgregori*	8.33%	0.69%	0.70%	0.07%

**FIGURE 2:**
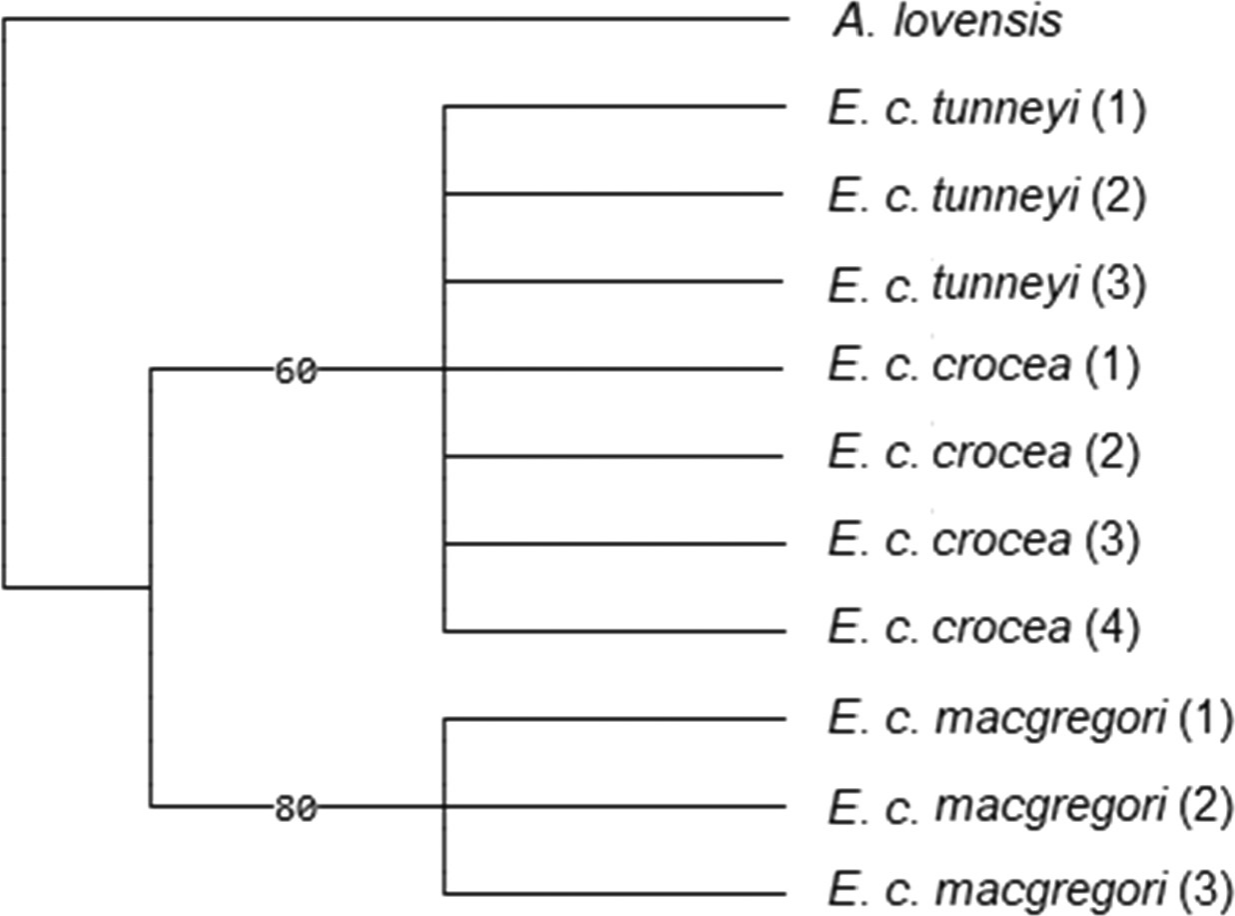
The maximum likelihood tree of three Yellow Chat (*Epthianura crocea*) subspecies (represented by each of the unique haplotypes for each subspecies) and the Gibberbird (*Ashbyia lovensis*) as an outgroup. The numbers on the branches indicate the bootstrap support values

A phylogenetic tree (Figure 2) of the three Yellow Chat subspecies and outgroup *Ashbyia lovensis* showed that the subspecies *E. c. macgregori* is supported as a sister group to *E. c. crocea* and *E. c. tunneyi*, but no such sister group is supported between *E. c. crocea* and *E. c. tunneyi*.

AMOVA results indicated that, of the total molecular variance present in the three Yellow Chat subspecies, 73% of that variance was between the subspecies, while the other 27% was within each subspecies (Table 3). Results show that the PhiPT value of 0.73 is highly significant (*p* = .001). We found a pairwise PhiPT ranging from 0.031 (*E. c. crocea* vs. *E. c. tunneyi*, *p* = .155) to 0.79 (*E. c. tunneyi* vs. *E. c. macgregori*, *p* = .001) and 0.84 (*E. c. crocea* vs. *E. c. macgregori*, *p* = .001). Overall, this demonstrates high genetic differentiation between subspecies, driven by the divergence of *E. c. macgregori* from *E. c. crocea* and *E. c. tunneyi* but no significant differentiation between *E. c. crocea* and *E. c. tunneyi*. Of the 11 unique haplotypes detected, haplotype and nucleotide diversity was higher for *E. c. tunneyi* and *E. c. macgregori* than for *E. c. crocea* (Table 1).

**TABLE 3:** Summary table of the analysis of molecular variance (AMOVA) both between and within three Yellow Chat subspecies (*p* = .001)

Source	*df*	SS	MS	Est. var.	%
Between subspecies	2	56.817	28.408	2.030	73%
Within subspecies	40	29.858	0.746	0.746	27%
Total	42	86.674		2.776	100%

## 4 DISCUSSION

This study represents the most comprehensive examination of the genetics of the Yellow Chat to date. It is the first study to include samples from multiple individuals of all three currently recognized subspecies. Analysis of mtDNA has been shown to reveal inconsistencies in the boundaries of subspecies in multiple bird taxa (Zink, 2004), and this may be the case in this study. We found that both the average pairwise differences between haplotypes and the pairwise PhiPT indicate that *E. c. crocea* and *E. c. tunneyi* have not been genetically isolated from one another very long, if at all, as there is no significant genetic divergence between them. Conversely, the genetic divergence that we found between *E. c. crocea* and *E. c. macgregori* suggests that the two subspecies have been genetically isolated for a considerable time, as was observed by Houston et al. (2015). We found low levels of both haplotype and nucleotide diversity in the cytochrome b sequences of all three subspecies, which may be a concern for the ongoing conservation of isolated populations.

### 4.1 Phylogeny

The lack of observed genetic divergence between *E. c. crocea* and *E. c. tunneyi* suggests that they have only recently separated and may, in fact, remain connected given that isolated populations develop increasingly divergent mtDNA over time (Weir & Schluter, 2008). Recent sightings of Yellow Chats of an unknown subspecies in the town of Katherine reduce the physical distance between known populations of the two subspecies considerably (Figure 1). Yellow Chats are reported to disperse long distances to exploit ephemeral habitat (Higgins et al., 2001), and *E. c. crocea* and *E. c. tunneyi* may disperse across the landscape as habitats become seasonally available. The coastal floodplains are seasonally inundated in the northern Australian wet season (December–May), which, to a ground‐foraging insectivore like the Yellow Chat, may provide an impetus to move elsewhere for more suitable, drier foraging habitat. Sightings of *E. c. tunneyi* are concentrated in the dry season (June–November; Armstrong, 2004, Kyne & Jackson, 2016), and their movements and behavior when the floodplains are inundated are poorly understood, partly as a result of the difficulty in surveying the floodplains when they are flooded. Regardless, there is potential for demographic connectivity between *E. c. crocea* and *E. c. tunneyi*, at least during extreme flood years when ground‐foraging on the coastal floodplains becomes difficult.

The amount of genetic divergence between *E. c. crocea* and *E. c. macgregori* suggests that the two subspecies have been isolated for a considerable time, although we note that our samples of *E. c. crocea* were from a location at almost maximum distance from the range of *E. c. macgregori*. Furthermore, all *E. c. crocea* samples were from a single location, whereas this subspecies has a wide inland range. Sampling of *E. c. crocea* across its range including in areas closest to *E. c. macgregori* would be desirable. The 0.7% average percentage pairwise difference between the two subspecies is similar to the genetic differences that were used to confirm the sub‐specific status of a range of other taxa (Penhallurick & Wink, 2004; Sackett et al., 2014; Zhou et al., 2004).

Land barriers are known to halt the dispersal of plants and animals (Eizirik et al., 2001; Evans et al., 2015; Lopes et al., 2013), and the Great Dividing Range, which sits between the ranges of *E. c. crocea* and *E. c. macgregori*, may limit contact between the two subspecies, especially given that almost all the habitat is heavily wooded (Neldner et al., 2017). *E. c. macgregori* has a small population size and density, which makes the likelihood of successful long‐distance dispersal low (Matthysen, 2005). Only small‐scale movements of approximately 10 km have been documented in *E. c. macgregori* (Houston, Elder, et al., 2018). Further, in a study of the genetic structuring of *E. c. macgregori* using microsatellites, there was no evidence of recent dispersal between two subpopulations of *E. c. macgregori* that were 140 km apart (Houston, Aspden, et al., 2018).

### 4.2 Genetic diversity

We found exceptionally low levels of both nucleotide and haplotype diversity in the mtDNA of the Yellow Chat (Table 1). For example, all three subspecies had less nucleotide and haplotype diversity in their cytochrome b gene than the Critically Endangered Magenta Petrel (*Pterodroma magenta*; Lawrence et al., 2008), the Vulnerable Southern Gray Shrike *Lanius meridionalis koenigi* (Padilla et al., 2015), and less nucleotide diversity than the Endangered San Clemente Loggerhead Shrike *Lanius ludovicianus koenigi* (Mundy et al., 1997), although the sample size in each of these studies was higher than for this study (117, 106, and 93, respectively). While more detailed genetic analysis using genome‐wide single nucleotide polymorphisms (SNPs) or whole genome sequencing would offer a more comprehensive dataset, mtDNA cytochrome b data are a useful tool to examine relative genetic diversity between species (Bowers et al., 1994; Zardoya & Meyer, 1996) and are frequently used in genetic analysis of birds (Momeni et al., 2022; Pârâu et al., 2019; Wang et al., 2016).

The low population size of both *E. c. macgregori* and *E. c. tunneyi* means that a low level of genetic diversity for these two subspecies is not surprising (Frankham, 1996). However, that it is *E. c. crocea* that has the lowest genetic diversity of the three subspecies is unexpected, as they have the largest population and widest distribution. This low genetic diversity in *E. c. crocea* may be due to sampling bias since all of the *E. c. crocea* samples were obtained on the same morning, in the same location, and appeared to be from individuals in the same flock. The individuals in the *E. c. crocea* samples may therefore be closely related, and broader sampling across this subspecies' extensive range would likely reveal greater genetic diversity. The sampling of both *E. c. macgregori* and *E. c. tunneyi* was over multiple days at multiple locations and, due to the low population sizes of these two subspecies, would represent a much larger proportion of the total population for those subspecies.

### 4.3 Phylogeography

Mitochondrial mutation rates have been used to date the separation of bird species and subspecies (Nabholz et al., 2009; Weir & Schluter, 2008). Rates in passerine birds are variable, but are estimated to average ~2.1% per million years for the cytochrome b gene (Weir & Schluter, 2008), although rates may be up to 10% per million years for some species (Nabholz et al., 2009). A genetic divergence of 0.43% has been calculated between *E. c. crocea* and *E. c. macgregori*, which was extrapolated to a separation period of ~215,000 years or less (Houston et al., 2015). Such a separation period incorporates two periods of glacial aridity and lends support to the theory of Pleistocene range expansion by the arid‐adapted Yellow Chat (Houston et al., 2015). We calculated a genetic divergence of 0.70% for *E. c. crocea* and *E. c. macgregori* using the same *E. c. macgregori* tissue but tissue from 8 *E. c. crocea* rather than the single sample used by Houston et al. (2015). This divergence equates to a separation period of ~350,000 years. While we calculated a genetic divergence of 0.05% for *E. c. crocea* and *E. c. tunneyi*, a calculation of their separation is not appropriate, as there was a similar amount of genetic divergence within the populations of *E. c. crocea* and *E. c. tunneyi*, and their pairwise population PhiPT of 0.031 was not significant (*p* = .155).

The freshwater wetlands on the floodplains that *E. c. tunneyi* inhabit are thought to have formed within the last 5000 years (Woodroffe et al., 1987). All known records of *E. c. tunneyi* are exclusively from these wetlands (Birdlife Australia, 2020; eBird, 2021; Higgins et al., 2001) suggesting that chats may have only dispersed to this area since the wetland formation. This study cannot confirm this hypothesis, as such a recent dispersal would likely constitute too short a timescale to be detected by mitochondrial mutation rates. However, evidence that Yellow Chats can disperse across the savanna woodlands that surround the freshwater wetlands is supported by the sightings of birds at Katherine, which is surrounded by savanna woodland. Another hypothesis is that *E. c. tunneyi* is descended from a population that dispersed there during more arid periods of the Quaternary, which were frequent before the Holocene (Nix & Kalma, 1972). This hypothesis has been used to explain the presence of isolated populations of arid‐evolved birds (e.g., Hooded Robin *Melanodryas cucullata melvillensis* on the Tiwi Islands: Woinarski et al., 2021). We cannot currently distinguish between these hypotheses because the most recent arid period was no more than 20,000 years ago (Miller et al., 2018; Nix & Kalma, 1972; Rowe et al., 2021).

### 4.4 Further research

The lack of significant divergence in the cytochrome b sequences of *E. c. tunneyi* and *E. c. crocea* raises questions about the subspecific status of *E. c. tunneyi*, particularly the geographical isolation that was used to separate them (Keast, 1958). This result needs to be interpreted cautiously, as there is evidence for mtDNA capture by congeneric species that are otherwise strongly characterized at a species level (Andersen et al., 2021). A more robust genetic examination beyond the level of a single locus (i.e., cytochrome b used here) would provide greater clarity of the genetic differences or similarities between *E. c. tunneyi* and *E. c. crocea* and if they are truly isolated genetically. The low quality and quantity of DNA recovered from the feathers of the Yellow Chats precluded the use of sequencing techniques such as double digest restriction‐site associated DNA sequencing (ddRADseq) and Diversity Arrays Technology sequencing (DArTSeq) (for example as in Battey & Klicka, 2017). Blood samples may provide higher quality and yield of DNA and should be considered for further work. Obtaining samples from more individuals from each subspecies and at different parts of their ranges (particularly for *E. c. crocea*) would also provide a more comprehensive picture of Yellow Chat phylogeny and genetic variation.

The taxonomic treatment of *E. c. tunneyi* is significant as its taxonomic validity is assumed in its listing as a threatened taxon (EPBC, 2006). The lack of significant difference in the cytochrome b sequences of *E. c. crocea* and *E. c. tunneyi*, coupled with recent Yellow Chat sightings that reduce the distance between their respective ranges, cast doubt upon their geographic isolation, one of the two criteria used to delineate these subspecies. Future taxonomic reclassification of *E. c. tunneyi* and *E. c. crocea* will need to examine reported plumage differences, which, along with geographic isolation, has been used to justify subspecific separation (Keast, 1958).

## AUTHOR CONTRIBUTIONS

**Robin Leppitt:** Conceptualization (lead); data curation (lead); formal analysis (lead); methodology (lead); visualization (equal); writing – original draft (lead); writing – review and editing (lead). **Alea M Rose:** Conceptualization (equal); data curation (lead); formal analysis (lead); methodology (lead); writing – original draft (equal); writing – review and editing (equal). **Wayne Houston:** Conceptualization (equal); data curation (equal); formal analysis (equal); methodology (equal); writing – original draft (equal); writing – review and editing (equal). **Peter Kyne:** Conceptualization (equal); data curation (equal); formal analysis (equal); methodology (equal); writing – original draft (equal); writing – review and editing (equal). **Sam Banks:** Conceptualization (equal); data curation (equal); formal analysis (equal); methodology (equal); writing – original draft (equal); writing – review and editing (equal). **John Woinarski:** Conceptualization (equal); data curation (equal); formal analysis (equal); methodology (equal); writing – original draft (equal); writing – review and editing (equal). **Stephen Garnett:** Conceptualization (equal); data curation (equal); formal analysis (equal); methodology (equal); writing – original draft (equal); writing – review and editing (equal).

## CONFLICT OF INTEREST

None declared.

## Data Availability

GenBank accession numbers: ON854511 ON854512 ON854513 ON854514 ON854515 ON854516 ON854517 ON854518 ON854519 ON854520 ON854521 ON854522 ON854523 ON854524 ON854525 ON854526 ON854527 ON854528 ON854529 ON854530 ON854531 ON854532 ON854533 ON854534 ON854535 ON854536 ON854537 ON854538 ON854539 ON854540 ON854541 ON854542 ON854543 ON854544 ON854545 ON854546 ON854547 ON854548 ON854549 ON854550 ON854551 ON854552 ON854553.
